# Root-associated microbial diversity and metabolomics in maize resistance to stalk rot

**DOI:** 10.3389/fmicb.2024.1468627

**Published:** 2024-12-12

**Authors:** Liming Wang, Jiao Jia, Qianfu Su, Hongzhe Cao, Shiqi Jia, Helong Si, Zhiyan Cao, Shujie Ma, Jihong Xing, Kang Zhang, Jingao Dong

**Affiliations:** ^1^State Key Laboratory of North China Crop Improvement and Regulation, Hebei Agricultural University, Baoding, China; ^2^Hebei Key Laboratory of Plant Physiology and Molecular Pathology, Hebei Agricultural University, Baoding, China; ^3^College of Plant Protection, Hebei Agricultural University, Baoding, China; ^4^Institute of Plant Protection, Jilin Academy of Agricultural Sciences, Changchun, China; ^5^Key Laboratory of Integrated Crop Pest Management in Northeast China, Ministry of Agriculture and Rural Affairs, Changchun, China; ^6^College of Life Sciences, Hebei Agricultural University, Baoding, China

**Keywords:** maize, stalk rot, microbial diversity, metabolomics, root microecology

## Abstract

As one of the three major food crops in the world, maize plays a significant role in alleviating the food crisis. Maize stalk rot can reduce maize yield and mechanical harvesting efficiency. In addition, mycotoxins such as Deoxynivalenol (DON) and Zearalenone (ZEN) produced by maize stalk rot pathogens can also harm livestock and human health. Maize stalk rot is an infection of the whole growth period, and there are no effective control measures at present. Therefore, it is of great significant to study the pathogenesis and control mechanism of stalk rot from multiple perspectives. In the present study, root and rhizosphere soil of disease-resistant inbred line Y853 and disease-susceptible inbred line Q478 were collected at the dough stage (R4) and maturity stage (R6) of maize, respectively. The effects of resistant/susceptible inbred line on soil microorganisms were analyzed by amplicon sequences and metabolomics. The results showed that there was different microbial community composition from different inbred lines in different growth stages. Specifically, the abundance of *Arthrobacter*, *Streptomyces* and *Bacillus* in R4 rhizosphere soil was higher than that of R6, while the rhizosphere fungal composition of LR853 was significantly different from that of the other three compartments. Co-occurrence network analysis showed that the pathogen *Fusarium* had the highest degree centrality and closeness centrality in the DR478. Moreover, metabolomics analysis showed that four main metabolic pathways were significantly enriched, and 15 metabolites were upgrade in resistant inbred line. Furthermore, microbes, especially fungi, also were related to these 15 metabolites. Our results revealed that maize resistance to stalk rot is closely related to root-associated microbiota and rhizospheric metabolites, which would be a new perspective of phytopathogenic biocontrol.

## 1 Introduction

Maize (*Zea mays* L.; corn) is a versatile multi-purpose cereal crop for human food/nutrient security, poultry demand, and fueling production (Erenstein et al., 2022). However, its annual yield is significantly decreased due to pests and pathogens (P&Ps), in which maize crop losses caused by *Fusarium* and *Gibberella* stalk rots is the highest among all diseases, with a global average of more than 4% (Savary et al., 2019). Additionally, *Fusarium* can produce fumonisins, which is highly dangerous for humans and animals’ consumption (Quesada-Ocampo et al., 2016; Liang et al., 2024). To overcome detrimental effect of the plant disease, several resistant cultivars and agrochemical pesticides were applicated (Shin et al., 2014). However, because the *Fusarium* and *Gibberella* stalk rots is infected during the whole growth period of maize, leading to challenges in effective chemical control. Additionally, the use of agrochemicals can harm beneficial microbiomes and impact human health or perception of agriculture (Geiger et al., 2010). With the vigorous promotion of straw returning to the field, high density planting and large use of nitrogen fertilizer, the accumulation of pathogens in the soil continues to increase, resulting in the huge disease pressure on the original variety resources, which cannot meet the agricultural application. In this regard, it is urgent to study the resistance mechanism of the newly bred high resistance inbred lines.

The interaction of microbial communities can inhibit the occurrence of soil-borne diseases, mainly including the following five aspects: antagonism and nutrient competition, parasitism, predation, induction of plant resistance, and interference with pathogenic signals of pathogens (Müller et al., 2016; Bai et al., 2022). Rhizosphere microorganisms, as the second genome of plants, contribute to plant nutrient absorption and resistance to adversity (Berendsen et al., 2012; Leach et al., 2017; Choi et al., 2021; Bai et al., 2022; Ling et al., 2022). The assembly and composition of plant rhizosphere microbiome are closely related to host plant–soil microbial community interaction (Badri et al., 2013; Bonito et al., 2014).

Host plants can regulate the root microbial structure, which in turn affects the growth and development of host plants (Guo et al., 2024). Studies have shown that many factors can affect the composition of plant microbial communities, which can be summarized as rhizosphere effects (effects of root metabolites on microbial communities), immune system, nutrient transfer, and host genotypic stress response (Müller et al., 2016; Bai et al., 2022). Among them, root metabolites (sugars, amino acids, organic acids, fatty acids, and secondary metabolites) are important forms of communication between plants and microorganisms, and can also attract microorganisms to transfer from the root circumference to the rhizosphere (Bulgarelli et al., 2013; Baetz and Martinoia, 2014). However, root metabolites directly affect rhizosphere soil state and thus affect rhizosphere microecology. The interaction between the characteristics of microbial substrate absorption and plant exudates is a molecular mechanism by which plants influence microbial communities through the regulation of root exudates (Zhalnina et al., 2018).

In addition, plants recruit beneficial microorganisms by altering root secretions (Busby et al., 2017; Pang et al., 2021; Wagner et al., 2021), which can inhibit the damage of pathogens by direct and indirect means (Mendes et al., 2011; Santhanam et al., 2015; Cha et al., 2016; Hu et al., 2016), including the secretion of antibacterial compounds (Chen et al., 2018; Helfrich et al., 2018), hyperparasitism (Parratt and Laine, 2018), and competition with pathogens for resources such as nutrients and space (Zelezniak et al., 2015). Therefore, it is of great significance to reveal the influence mechanism of plant root exudes on the composition and assembly of rhizosphere microbial communities.

Here, maize stalk rot resistant inbred line Y853 and susceptible inbred line Q478 were used as research materials to analyze the root and rhizosphere microbial communities in the douph and maturity stages. LC–MS technology was used to analyze root metabolites, and the relationship between key rhizosphere microorganisms and key root metabolites was analyzed. This study provided a theoretical basis for elucidating the relationship between rhizosphere microbial community and resistance to *Fusarium* infection in maize stage, and laid a foundation for green prevention and control of maize stalk rot.

## 2 Materials and methods

### 2.1 Sampling location and samples collection

This study was conducted in the field of Gongzhuling City, Jilin province, one of the major corn planting areas in China. The sampling time was douph stage (R4, approximately 26–30 days after the first silks to emerge) and maturity stage (R6, approximately 50–60 days after the first silks to emerge) of maize. At each sampling site, three quadrats were randomly established. For root sampling, three resistant inbred lines plants and three susceptible inbred lines plants were randomly selected from each quadrat, and roots were removed with a shovel, shaken to remove loosely adhered soil, and clipped and then immediately placed in a sterile bag. Rhizosphere soil from the same plant was collected afterward. The litter and soil around the roots were removed using a shovel, and the soil attached to the roots was collected and placed in a 50 mL sterilized centrifuge tube (Edwards et al., 2015). Samples from different sample points were mixed and numbered and brought back to the laboratory in an ice box. For the root sample collection, the root was vigorously shaken to remove the adhesive soil, and the root was shaken in a 500 mL sterile triangle bottle containing 50 mL PBS for 20 min. The root was repeatedly washed twice in PBS, and the remaining adhesive microorganisms were removed by ultrasound (Beirinckx et al., 2020).

### 2.2 DNA extraction and PCR amplification

According to standard protocols, soil DNA was extracted using the E.Z.N.A.® Soil DNA Kit (OMEGA, USA), while root material was extracted using the FastDNA Spin Kit (MP Biomedicals, Solon, OH, USA). The DNA concentration was measured with a Nanodrop 2000 (Thermo Scientific, USA) and assessed by agarose gel electrophoresis. DNA was stored at −80°C until further analysis. The V4–V5 bacterial 16S rRNA gene regions were amplified with the primers 338F 5′-ACTCCTACGGGAGGCAGCA-3′ and 806R 5′-GGACTACHVGGGTWTCTAAT-3′ (Xu et al., 2016), the primers for the 16S V5–V7 rRNA gene region were 799F 5′-AACMGGATTAGATACCCKG-3′ and 1193R 5′-ACGTCATCCCCACCTTCC3′ (Cui et al., 2023), and the ITS region of the fungal rRNA gene was amplified using the fungal-specific primer pair ITS1F (5′-CTTGGTCATTTAGAGGAAGTAA-3′)/ITS2R (5′-GCTGCGTTCTTCATCGATGC-3′) (Adams et al., 2013). Polymerase chain reactions (PCRs) were performed as follows: 3 min initial denaturation at 95°C, 27 cycles of denaturation at 95°C (30 s), annealing at 55°C (30 s), elongation at 72°C (45 s), and final extension at 72°C for 10 min. After purifying the amplicons and quantification, an Illumina Miseq platform was used for sequencing.

### 2.3 High throughput sequencing and analysis

The basic raw data analysis procedures were performed by using Majorbio Cloud Platform.^1^ Besides, in this study, the resulting paired sequence reads were then merged, trimmed, filtered, aligned, and clustered using operational taxonomic unit (OTU) using USEARCH v. 5.2.236 software. Sequences with ≥97% similarity were assigned to the same OTU using the UPARSE-OTU algorithm in QIIME. Principal component analysis (PCA) and the distance-based redundancy analysis (RDA) were conducted in R v.3.2.1 with the vegan package (Oksanen et al., 2022).

### 2.4 Network analysis

Bacterial and fungal networks were constructed using the Majorbio Cloud Platform (see text footnote 1) in order to understand how rhizosphere soil and root endophytic microbial interactions change in different inbred lines. Nodes and edges in the network represent the genera of bacteria and fungi, as well as their correlations with each other, respectively. Genera with a relative abundance of less than 0.01% are filtered due to their poorly representative (Ma et al., 2016). Only when the absolute value of the spearman correlation coefficient is greater than or equal to 0.7 and the *p*-value is less than 0.01, it is considered to be a valid correlation in the network. The topological features of the network are mainly measured by Degree, Closeness Centrality, Betweenness Centrality and Degree Centrality. The networks of rhizosphere soils and endophytes were graphically showed in Gephi 0.10.^2^

### 2.5 Soil metabolite analysis

Take 50 mg of rhizosphere soil into 2 mL centrifugal tube and add a 6 mm diameter grinding bead. Extraction solution (400 uL, methanol: water = 4:1 (v:v)) containing 0.02 mg/mL of the internal standard (L-2-chlorophenylalanine) was used for metabolite extraction. Samples were ground by the Wonbio-96c (Shanghai wanbo biotechnology co., LTD) frozen tissue grinder for 6 min (−10°C, 50 Hz), followed by low-temperature ultrasonic extraction for 30 min (5°C, 40 kHz). The samples were left at −20°C for 30 min, centrifuged for 15 min (4°C, 13,000*g*), and the supernatant was transferred to the injection vial for LC–MS/MS analysis. As a part of the system conditioning and quality control process, a pooled quality control sample (QC) was prepared by mixing equal volumes of all samples. The QC samples were disposed and tested in the same manner as the analytic samples. It helped to represent the whole sample set, which would be injected at regular intervals (every 5–15 samples) in order to monitor the stability of the analysis.

Principal component analysis (PCA) and orthogonal least partial squares discriminant analysis (OPLS-DA) was conducted by the R package “ropls” (Version 1.6.2). The metabolites with VIP > 1, *p* < 0.05 were determined as significantly different metabolites based on the Variable importance in the projection (VIP) obtained by the OPLS-DA model and the *p*-value generated by student’s t-test. The correlation and visualization between differential microbes and metabolites were conducted using the Majorbio Cloud Platform (see text footnote 1).

## 3 Results

### 3.1 Microbial diversity of rhizosphere and root-endophytic samples

A total of eight composite samples, including four rhizosphere soil and four endophytic samples (with three biological replicates per sample), were subjected to high-throughput sequencing using the Illumina MiSeq system. The 16S rRNA and ITS gene sequencing yielded 1,433,190 and 1,550,958 effective tags after quality control, in which, 643,090 and 829,308 reads were from rhizosphere soil bacteria and fungi, 790,100 and 721,650 reads were from endophytic bacteria and fungi, respectively. Rarefaction analyses showed that the curves of bacterial 16S genes and fungal ITS genes in both rhizosphere and endophytic tended to approach the saturation plateau (Supplementary Figure S1). Therefore, the sequencing depth was adequate for assessing the diversity of bacterial and fungal communities of our samples.

**Figure 1:**
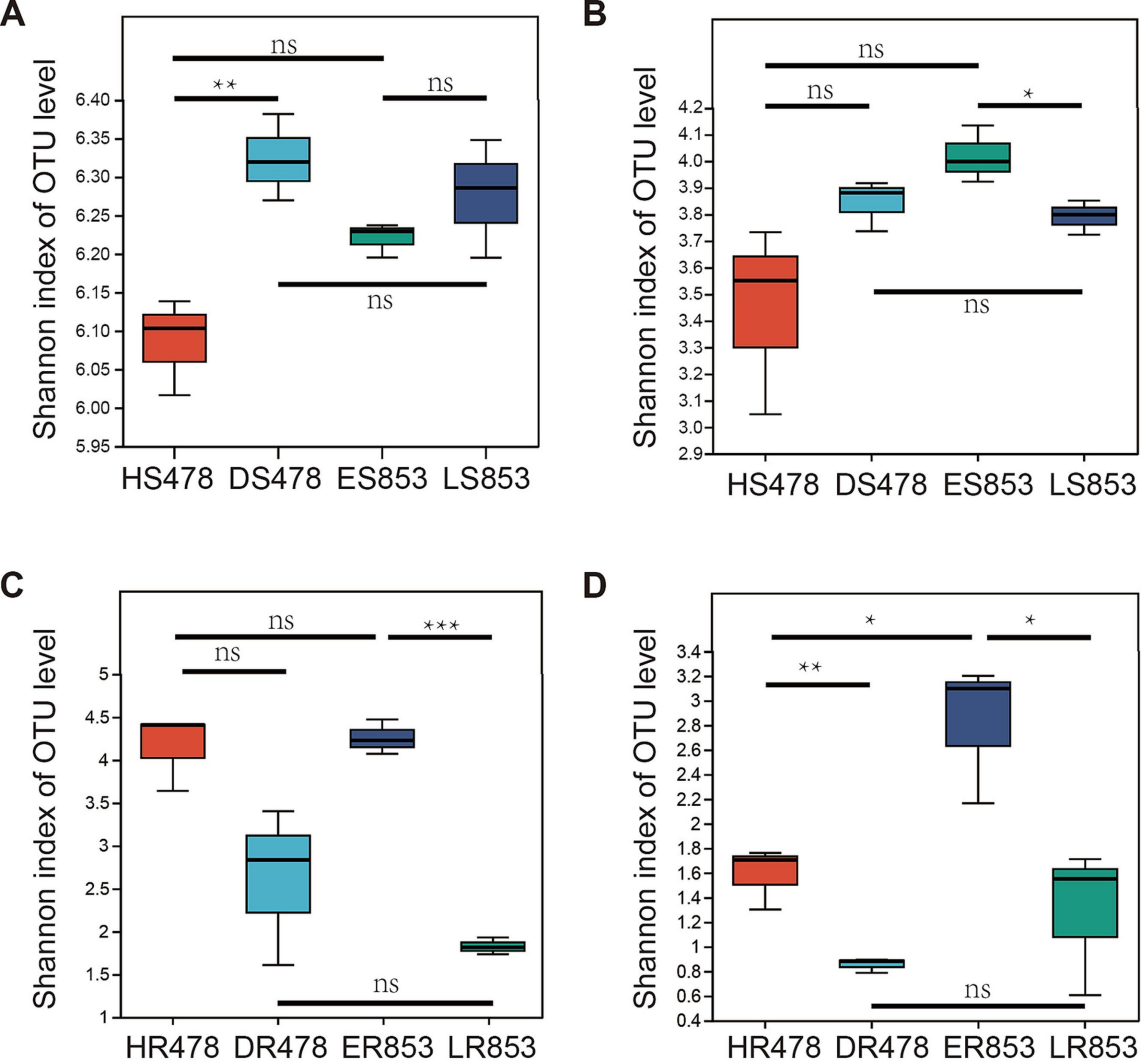
Diversity of the 16S rRNA gene-based bacterial communities and ITS gene based fungal communities. **(A)** Rhizosphere soil bacterial community; **(B)** rhizosphere soil fungal community; **(C)** root-endophytic bacterial community; **(D)** root-endophytic fungal community.

Alpha diversity indexes were calculated based on OTU level to quantify the diversity and richness of the microbial community. For rhizosphere soil bacteria, DS478 is significantly higher than HS478, but the bacterial diversity of resistant inbred lines in the two periods was not significantly different, and the bacterial diversity of different inbred lines in the same period was not significantly different (Figure 1A). According to the student’s t-test, there was no significant difference between the fungal diversity of HS478 and DS478, and no significant difference between the fungal diversity of different inbred lines in the same period, but only LS853 was significantly higher than ES853 (Figure 1B). For the root endophytic microbiome, there was no significant difference between HR478 and DR478 in bacterial community, while HR478 has higher Shannon index than DR478 in root-endophytic fungi community. ER853 and LR853 are significantly different with each other in root-endophytic microbial (bacterial and fungal) community (Figures 1C,D).

Despite the dynamics of microbial communities over the growing stage and healthy condition, there were also some stabilities of root-associated microbial members. We identified 2,012 stable bacterial OTUs and 343 stable fungal OTUs in the rhizosphere compartment (Supplementary Figure S2A,C). Among these 2,012 stable bacterial OTUs, nine OTUs accounted for more than 1% of the total. These nine OTUs belonged to three phylum *Actinomycetota*, *Pseudomonadota*, and *Chloroflexota*, which accounted for 17.51% in the stable bacterial OTUs (Supplementary Figure S2B; Supplementary Table S1). 25 fungal OTUs were more than 1% of the total and accounting for 72.13% in the 343 stable fungal OTUs, and these OTUs belong to *Basidiomycota*, *Ascomycota*, and *Mortierellomycota* (Supplementary Figure S2D; Supplementary Table S1). For the root-endophytic compartment, there were 117 stable bacterial OTUs and 36 stable fungal OTUs (Supplementary Figure S2E,G). 23 bacterial OTUs, accounted for more than 10% of the total, were accounting for 82.78%, and these OTUs belong to two phylum *Actinomycetota* and *Pseudomonadota* (Supplementary Figure S2F; Supplementary Table S1). Nine OTUs of root-endophytic fungi were more than 1%, and these OTUs were accounting for 94.99%, in which, OTU200 (*Exophiala*) accounts for 56.18% (Supplementary Figure S2H; Supplementary Table S1). Furthermore, *Sphingomonas*, *Tausonia*, *Myrmecridium*, *Fusarium*, *Sarocladium*, *Exophiala*, and *Setophoma* were shared with root and rhizosphere compartments.

### 3.2 Community composition based on the 16S rRNA gene (bacteria) or ITS gene (fungi)

**Figure 2:**
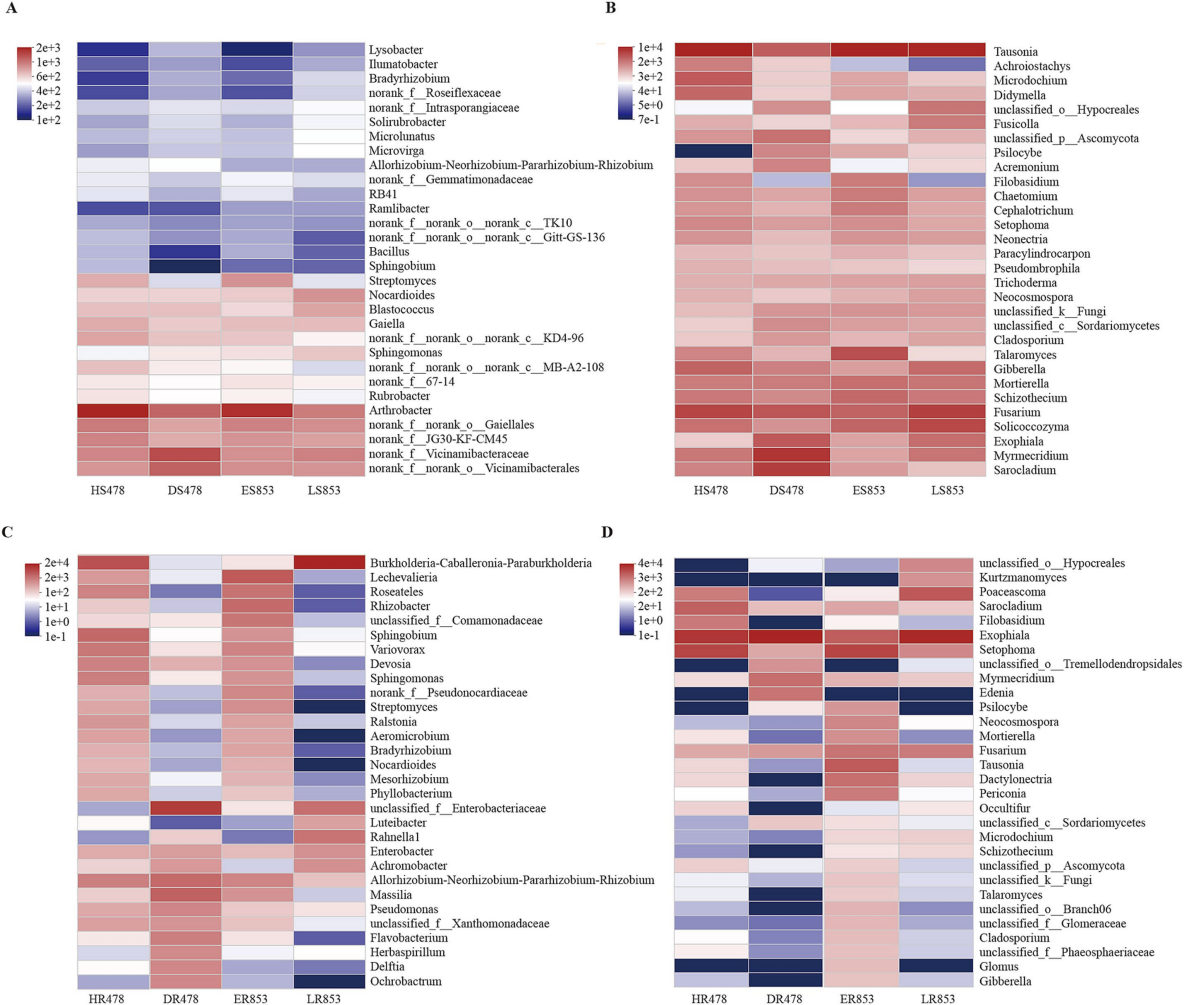
Heatmap of genus level for different treatments in different compartments. **(A)** Rhizosphere soil bacterial community; **(B)** rhizosphere soil fungal community; **(C)** root-endophytic bacterial community; **(D)** root-endophytic fungal community.

For bacterial composition (Figures 2A,C), the same growth stage had great similarity, especially in the Dough (R4) stage (HS478, ES853). In the rhizosphere soil, *Bacillus* and *Arthrobacter* were more abundant in the R4 stage than in the Maturity (R6) stage. In the root endophyte, there was a great difference in the composition of resistant inbred lines and susceptible inbred lines at R6 stage. In terms of the composition of fungi, both in the rhizosphere soil and root tissue, there was a large difference, and the abundance of endophytes was significantly lower, among which *Fusarium* had a large abundance (Figures 2B,D). The above results were also shown at the phylum level (Supplementary Figure S3). Specifically, the dominant 10 bacterial phyla with high abundances in the total soil samples were *Actinomycetota*, *Pseudomonadota*, *Acidobacteriota*, *Chloroflexota*, *Bacteroidota*, Gemmatimonadota, Myxococcota, *Bacillota, Candidatus Patescibacteria*, and *Methylomirabilota*. However, there was no significant difference in top five bacterial phyla among different compartments. *Bacillota*, *Gemmatimonadota*, and *Methylomirabilota* showed that R4 stage and healthy maize had higher relative abundance (Supplementary Table S2).

Variations in the microbial community across the four groups were evaluated by PCoA (Supplementary Figure S4). The soil bacterial communities significantly differed between inbred lines (Supplementary Figure S4A; ANOSIM *p* = 0.002, *R*^2^ = 0.5586), while the soil fungal communities significantly differed between diseased and healthy plants (Supplementary Figure S4B; ANOSIM *p* = 0.001, *R*^2^ = 0.7172). However, the endophytic bacterial communities had significantly different between diseased and healthy plants (Supplementary Figure S4C; ANOSIM *p* = 0.001, *R*^2^ = 0.8200). For the endophytic fungi communities, ER853 were significantly differed from LR853, and HR478 are significantly differed from DR478 (Supplementary Figure S4D; ANOSIM *p* = 0.001, *R*^2^ = 0.7659).

### 3.3 Microbiome assembly from rhizosphere soil to root endophyte

**Figure 3:**
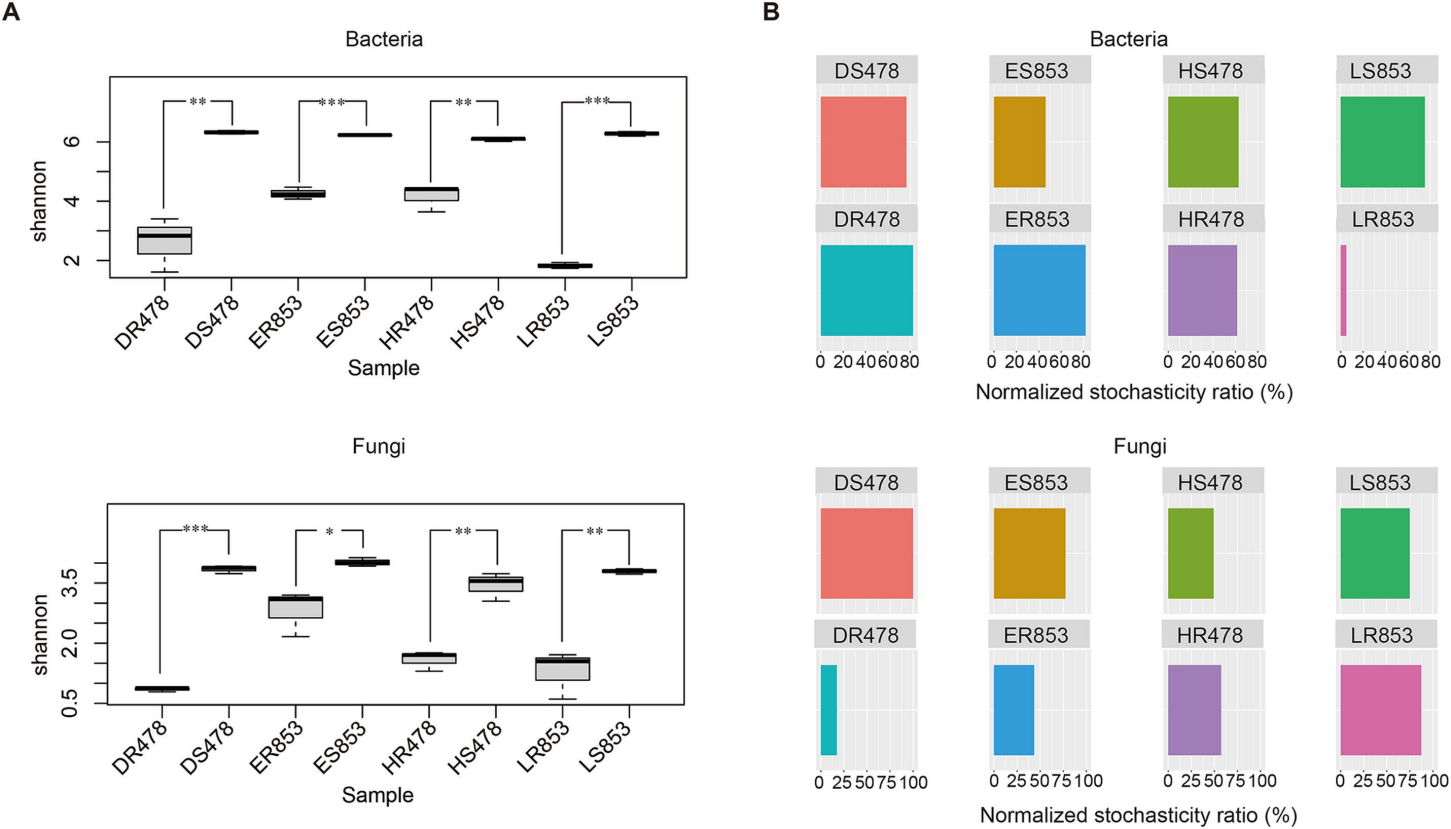
Correlation between rhizosphere microbial communities and root exudates. **(A)** Bacteria; **(B)** Fungi. The vertical axis represents different metabolites. The horizontal axis represents the different genera of microorganisms. * Represents significance (*p* < 0.05), ** represents significance (*p* < 0.01), *** represents significance (*p* < 0.001).

The diversity of bacteria and fungi in rhizosphere soil is significantly higher than that of root endophytes in both disease-resistant and disease-susceptible inbred lines (Figure 3A). This may be due to the host’s certain selectivity for microorganisms in the rhizosphere, which reduces the diversity of root endophytes. In addition, bacterial diversity was higher than fungal diversity in the same treatment. The NST ratio was calculated to evaluate the ecological stochasticity of microbial communities (Figure 3B). In this study, both rhizosphere bacteria and fungi communities were more strongly driven by stochastic processes (NST > 50), while root endophytic bacterial communities of LR853 exhibited a high deterministic ration, and root endophytic fungal communities of DR478 were driven by deterministic assembly processes (NST < 50%). Results indicated that the root endophytic microbial communities were more strongly driven by deterministic assembly processes in the R6 stage.

**Figure 4:**
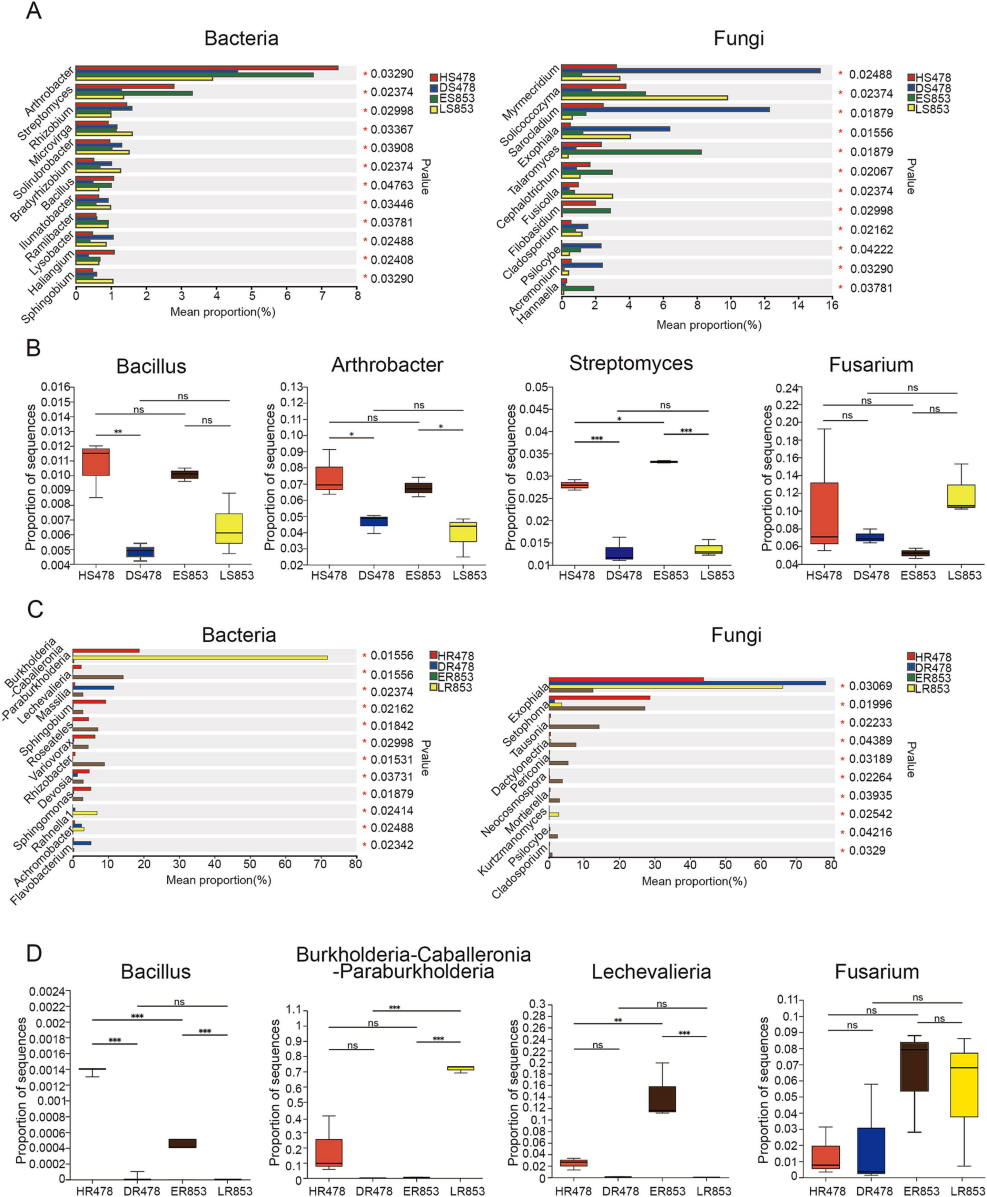
Relative abundances of genera that showed significant differences among samples. **(A)** Rhizosphere soil; **(B)** Bacillus, Arthrobacter, Streptomyces and Fusarium in rhizosphere soil; **(C)** root endophyte; **(D)** Bacillus, Burkholderia-Caballeronia-Paraburkholderia, Lechevalieria and Fusarium in root endophyte.

The bacteria and fungi with significant differences in relative abundance in rhizosphere soil and root endophyte were different (Figures 4A,C). *Bacillus*, a genus with extensive antagonistic effect against *Fusarium*, has a significantly higher relative abundance in rhizosphere soil in healthy plants than diseased plants. The relative abundance of *Arthrobacter* and *Streptomyces* was significantly higher at R4 stage compared with R6 stage. *Fusarium* has a certain relative abundance in rhizosphere soil, but there was no significant difference in each compartment (Figure 4B). The relative abundance of *Bacillus* was significantly higher in susceptible inbred lines of R4 (HR478) than resistant inbred lines of ER853 in root endophyte, while there was no significant difference between resistant inbred lines of R6 (almost 0). The results showed that the R4 stage of the same inbred line was significantly higher than the R6 stage. Moreover, the relative abundance of *Burkholderia* and *Lechevalieria* in infected plants were very low. The relative abundance of *Fusarium* in roots did not significantly differ among the samples (Figure 4D).

### 3.4 Microbial networks

**Figure 5:**
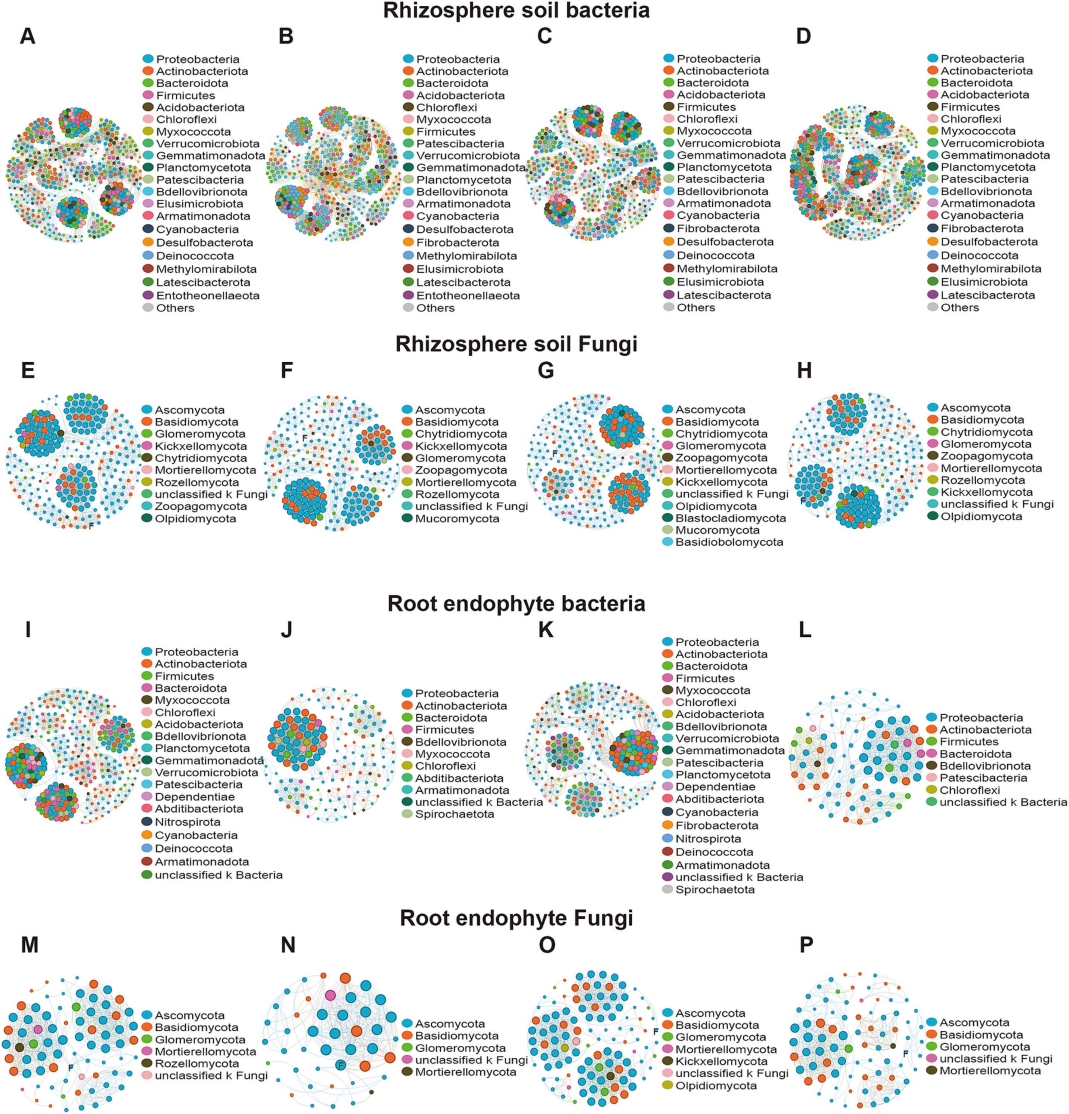
Co-occurrence network showing the co-occurrence patterns within the rhizosphere soil and root endophytic microbiomes of maize in different planting growth stages. **(A,E,I,M)**: H478; **(B,F,J,N)**: D478; **(C,G,K,O)**: E853; **(D,H,L,P)**: L853.

Microbial networks were established based on significant correlations (Pearson’s correlation, *p* < 0.05) among different treatments. Our results showed that the rhizosphere microbial network had more nodes and edges than the corresponding root microbial network. Bacterial networks have more nodes and edges than fungal networks in the same compartment (Figure 5). For rhizosphere, bacteria and fungi in different inbred lines at different growth stages had little change in nodes and edges (Figures 5A–H). In root endophyte, LR853 had the fewest nodes and edges in the bacterial network, while DR478 had the fewest nodes and edges in the fungal network (Figures 5I–P). LR853 had nearly twice as many nodes in the fungal network as DR478. Degree centrality is the most direct measure to describe node centrality in network analysis. The greater the degree of a node, the higher the degree centrality of the node, and the more important the node is in the network. The greater the closeness coefficient, the closer the node is to the center of the network. Through the analysis of the topological characteristics of *Fusarium* in the network, it can be seen that the degree centrality and closeness centrality in the root endophyte of DR478 was the highest.

### 3.5 Metabolic differences in maize rhizosphere soil across different inbred lines

**Figure 6:**
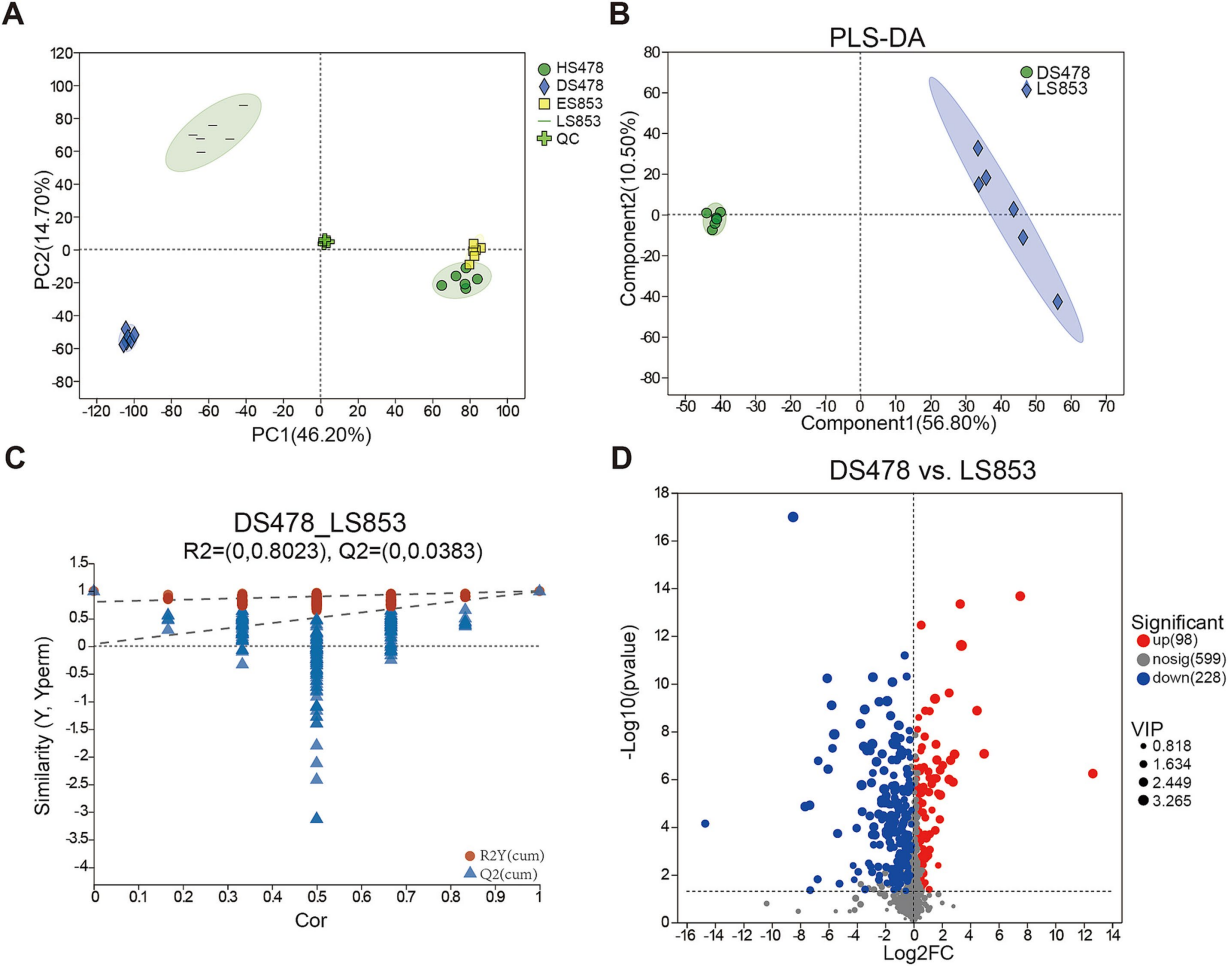
Metabonomic analysis of rhizosphere soil in different compartments. **(A)** Principal component analysis (PCA) of metabolites; **(B)** PLS-DA analysis of metabolites between DS478 and LS853. **(C)** Cross-validation model of PLS-DA. **(D)** The expression volcano map of differential metabolites up and down regulate.

To determine the response of soil metabolic activities to different inbred lines, 24 soil samples were analyzed using metabolomics on the UPLC-MS/MS platform. After quality control, PCA was used to analyze differences among four treatment samples (Figure 6A). The QC samples clustered together, suggesting that the method was stable and yielded high-quality data. Samples for the same treatments clustered and different treatment were separated from each other, indicating differences among five treatments. The dimensions P1 and P2 were able to explain 60.90% of observed differences. Totally, we detected 925 known metabolites from 24 soil samples, including lipids and lipid-like molecules (41.97%), organic oxygen compounds (14.48%), organoheterocyclic compounds (13.15%), organic acids and derivatives (11.55%), phenylpropanoids and polyketides (10.36%), benzenoids (4.12%), nucleosides, nucleotides, and analogs (1.99%), organic nitrogen compounds (0.40%), hydrocarbon derivatives (0.13%), hydrocarbons (0.13%), and organooxygen compounds (0.13%), and there were some metabolites were not classified (1.59%).

Multivariate statistical methods were conducted to analyze the high degree of inter-group correlation. The differences within and between the disease-susceptible inbred lines (DS478) and disease-resistant inbred lines (LS853) were analyzed using the supervised discriminant analysis statistical method PLS-DA model, so as to obtain the impacts of disease resistant/susceptible inbred lines on soil metabolites. According to PLS-DA (Figure 6B), there was similar metabolite composition across all treatments, but there were differences between the two inbred lines. The PLS-DA model indicated that the model was good and could be used to screen for DMs (R2Y = 0.8023, Q2Y = 0.0383) (Figure 6C). DMs were screened through volcano plots (Figure 6D). The result showed that there were 98 metabolites up-regulation and 228 metabolites down-regulation.

**Figure 7:**
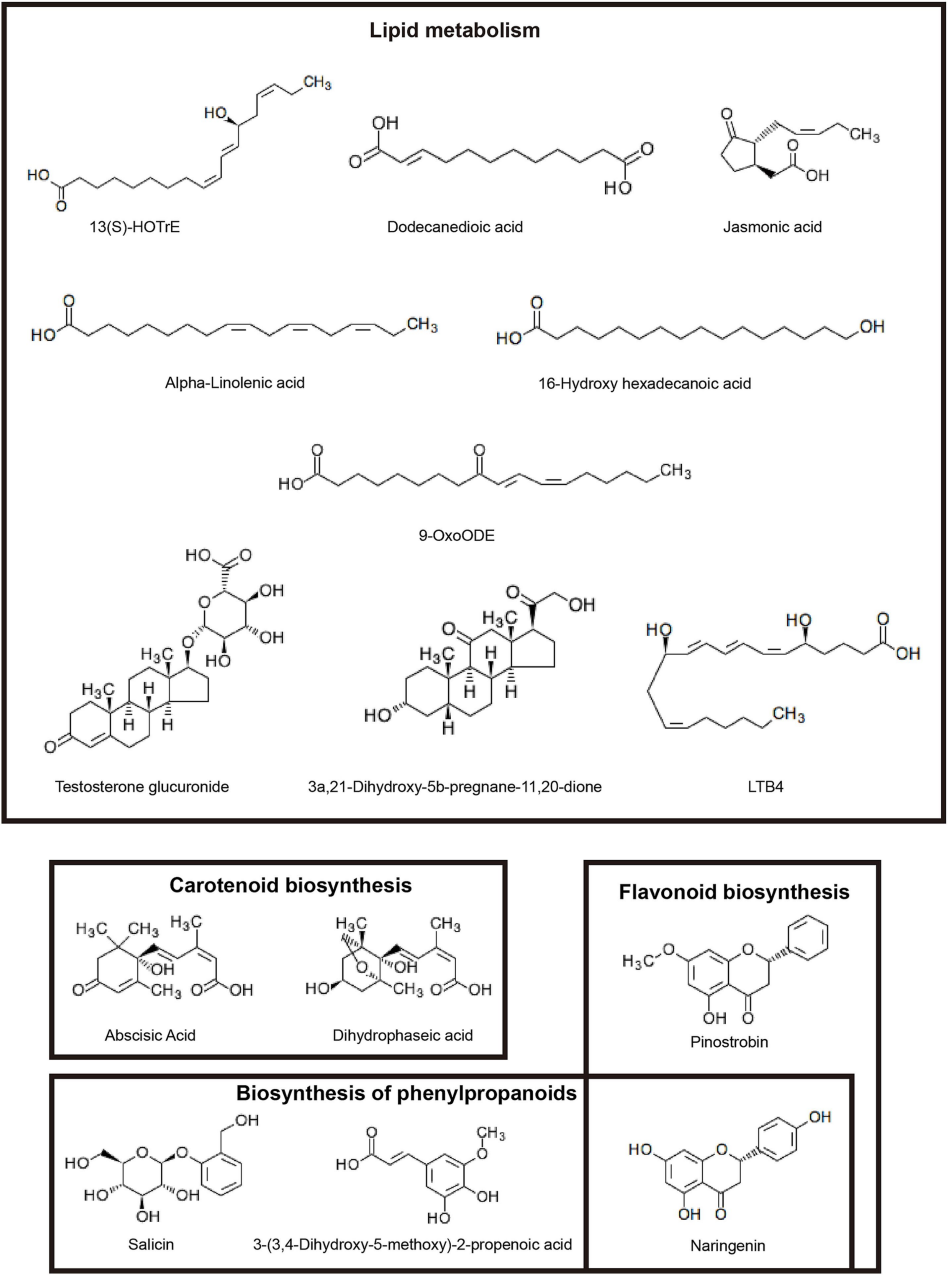
Structural formula of 15 differential metabolites.

To further explore metabolism affected by different inbred lines in rhizosphere soil, we found out 15 DMs by searching the KEGG pathway database (Figure 7). These metabolites were involved in carotenoid biosynthesis, flavonoid biosynthesis, biosynthesis of secondary metabolites, biosynthesis of phenylpropanoids, steroid hormone biosynthesis, arachidonic acid metabolism and cutin, suberine and wax biosynthesis, and so on.

### 3.6 The relationship between soil metabolites and microorganisms

**Figure 8:**
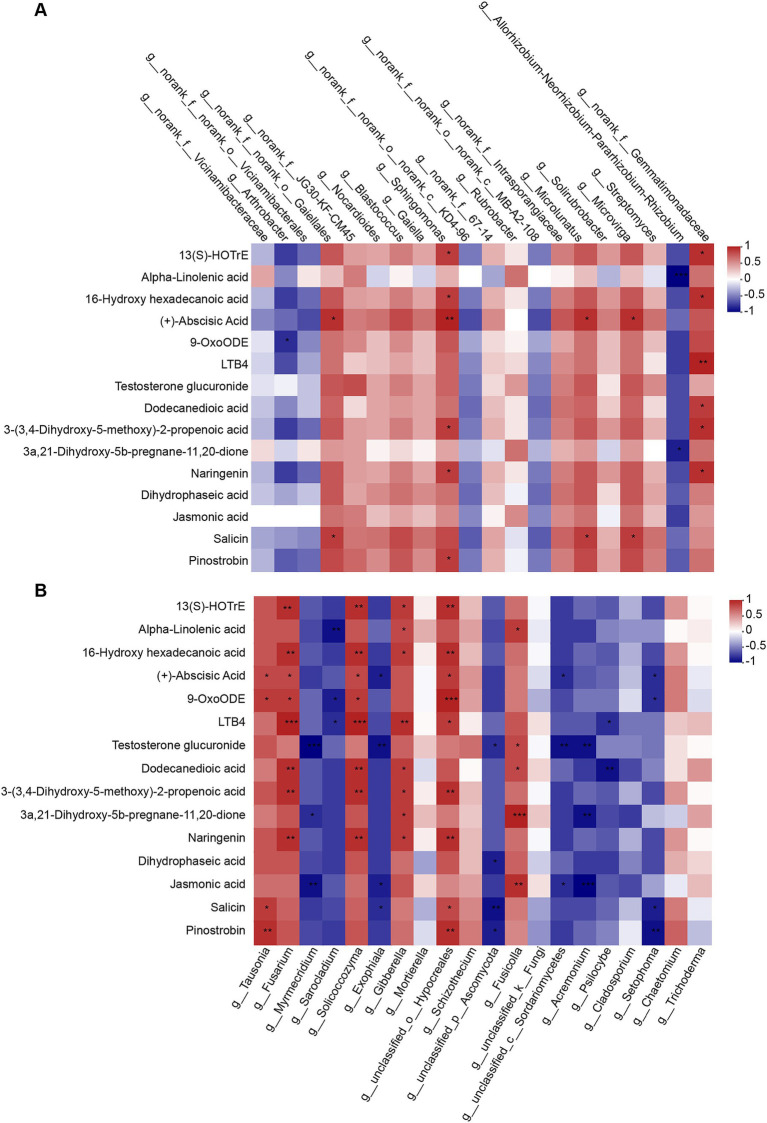
The diversity and stochasticity of microbial communities in rhizosphere soil and root endophyte for different treatments in different compartments. **(A)** Shannon indexes. Asterisks indicate significant differences among plant microhabitats, based on student’s *t*-test; **(B)** The normalized stochasticity ratio (NST) of bacterial and fungal communities under different plant compartments developed based on Jaccard distances with 50% as the boundary point between more deterministic (<50%) and more stochastic (>50%) community assembly.

Elucidating the relationship between microbes and their metabolites is a necessary step for researching mechanisms of plant-microbiome interactions. Therefore, we analyzed correlations between microorganisms and metabolites based on abundances. Significant (*p* < 0.05) and highly significant (*p* < 0.01) correlations were observed linking the top 20 bacterial (Figure 8A) and fungal (Figure 8B) genera with DMs. We can find that the relationship between bacteria and metabolites can be divided into two categories, one is negative correlation, such as *Arthrobacter*, *Allorhizobium-Neorhizobium-Pararhizobium-Rhizobium*, *Vicinamibacterales, KD4-96*, and *MB-A2-108*, where *Arthrobacter* was significantly negative correlation with 9-OxoODE. *Allorhizobium-Neorhizobium-Pararhizobium-Rhizobium* showed a significant negative correlation with Alpha-Linolenic acid and 3a, 21-Dihydroxy-5B-pregnane-11, 20-DiOne. And the other is positive correlation, in which *Gaiellales*, *Microlunatus* have the same correlation with 15 DMs, were significant positive correlation with Abscisic Acid and Salici. *Sphingomonas* positively correlated with 13(S)-HOTrE, 16-Hydroxy hexadecanoic acid, Abscisic Acid, 3-(3,4-Dihydroxy-5-methoxy)-2-propenoic acid, Naringenin and Pinostrobin. *Gemmatimonadaceae* positively correlated with 13(S)-HOTrE, 16-Hydroxy hexadecanoic acid, LTB4, Dodecanedioic acid, 3-(3,4-Dihydroxy-5-methoxy)-2-propenoic acid and Naringenin.

Relationships between fungi and metabolites are shown in Figure 8B, which were more than those between bacteria and metabolites. The *Fusarium* was significantly positively correlated with 13(S)-HOTrE, 16-Hydroxy hexadecanoic acid, Abscisic Acid, 9-OxoODE, LTB4, Dodecanedioic acid, 3-(3,4-Dihydroxy-5-methoxy)-2-propenoic acid and Naringenin, and *Solicoccozyma* has the same correlation with these DMs. *Gibberella*, the sexual state of *Fusarium*, was positively correlated with 13(S)-HOTrE, Alpha-Linolenic acid, 16-Hydroxy hexadecanoic acid, LTB4, Dodecanedioic acid, 3-(3,4-Dihydroxy-5-methoxy)-2-propenoic acid, 3a,21-Dihydroxy-5b-pregnane-11,20-dione and Naringenin. These results showed that the relative abundances of two bacterial genera (*Sphingomonas* and *Gemmatimonadaceae*) and five fungal genera (*Fusarium*, *Solicoccozyma*, *Gibberella*, *Hypocreales*, and *Fusicolla*) had the strongest correlations with certain metabolites, which suggest that these microorganisms may be involved in the formation of most metabolites in soil.

## 4 Discussion

### 4.1 Microbiome assembly from rhizosphere soil to root endophyte

The results of the Shannon index showed that the microbial diversity of different habitats under the same treatment was higher in the rhizosphere than in the root endophyte (Figure 1), which was the same as the results of many studies (Cui et al., 2023; Ji et al., 2023). In theory, deterministic processes drive ecological communities to be more similar stochastic processes (Pontarp and Petchey, 2016). We could assume that there was a deterministic process from rhizosphere soil to root-endophyte. To verify this hypothesis, we further analyzed the assembly process of rhizosphere soil to root endophyte microorganisms. According to the comparison of diversity, the microbial diversity of rhizosphere soil was significantly higher than that of root tissue in both 478 and 853 (Figure 3A). Normalized stochasticity ratio (NST) measures the relative importance of randomness versus determinism, taking into account situations in which deterministic factors drive communities that are more similar or different than a random model would expect (Ning et al., 2019). Through NST analysis, it was found that the root tissue of resistant inbred line 853 had a very strong orientation to bacteria, while the root tissue of susceptible inbred line 478 had a very strong orientation to fungi, both of which occurred at the R6 stage (Figure 3B). We can conclude that this directional selection may be related to the disease resistance of plants. Resistant inbred lines select specific bacterial communities to enter the plant tissues to help resist the infection of pathogens, while susceptible inbred lines have no directional selection ability on bacteria, but only have strong directional selection ability on fungi.

### 4.2 Maize resistance is related to the importance of pathogens in root communities

The results of different microorganisms showed that there were great changes from rhizosphere to root endophyte. The proportion of *Bacillus* was higher in healthy plants than in diseased plants, and higher in R4 stage than in R6 stage (Figure 4). Although *Bacillus*, a plant growth-promoting bacteria (PGPB), has been reported to control fungal phytopathogens in many plant species (Pieterse et al., 2014; Zhang K. et al., 2022; Dobrzynski et al., 2023), our results showed that the proportion of *Bacillus* would not directly affect disease resistance of plants, and soil or root tissues of R4 stage can be considered for higher success rate when separating and screening antagonistic *Bacillus*, which need more experiments to prove. According to the results of *Fusarium* analysis, there was no significant difference in either rhizosphere or root endophyte, indicating that the number of pathogens was not the only factor determining the disease susceptibility and resistance of plants. The results of network analysis show that bacterial networks were more complex than fungal networks, and the differences of bacterial networks in the rhizosphere were not large, while there were large changes in the rhizosphere at different periods (Figure 5). The degree centrality and closeness centrality of pathogenic *Fusarium* were the largest in the diseased roots endophyte, that is, *Fusarium* played a key role in the community stability in the roots of infected plants. It can be assumed that disease resistance may be related to the importance of pathogen in the community. In disease control, especially soil-borne disease control, we can consider destroying the importance of pathogenic bacteria in the community structure rather than just killing pathogenic bacteria as a breakthrough.

### 4.3 Rhizosphere metabolites are important drivers of microbial community structure

Soil metabolites come mainly from plant roots and microorganisms. Root exudates stimulate and recruit soil microorganisms as the first line of defense against soil-borne diseases (Trivedi et al., 2020; Ma et al., 2023). In this study, two maize inbred lines significantly changed the metabolite spectrum. Resistant inbred line LS853 caused most metabolites to be up-regulated (228) (Figure 6). The results indicated that plant disease resistance is an important factor affecting microbial activity and root exudates. We identified metabolites that are significantly up-regulated in disease-resistant inbred lines and are associated with plant disease resistance. They belong to four metabolic pathways, namely Lipid metabolism, carotenoid biosynthesis, flavonoid biosynthesis and biosynthesis of phenylpropanoids. Lipids are important cellular components with structural, storage, signaling, and defense functions. During plant-pathogen interactions, lipids play a role in pathogen-induced local and systemic immune responses. They interact with various components of the plant immune network and can both positively and negatively regulate plant defenses (Kuźniak and Gajewska, 2024). Our study showed that four metabolites (13(S)-HOTrE, 16-Hydroxy hexadecenoic, LTB4, and Dodecanedioic acid), related lipid metabolism pathway, were significantly positive corelated with the pathogen *Fusarium.* These metabolites may help hosts recognize the pathogen’s invasion (Cavaco et al., 2021).

Rhizosphere metabolites are important drivers of microbial community structure (Bourceret et al., 2022; Cheng et al., 2022; Zhang J. et al., 2022; Li et al., 2023; Ma et al., 2023). The change of microbial composition determines the change of soil metabolites to a certain extent, and then determines the circulation and metabolism of nutrients in the soil. Therefore, it is of great significance to reveal the correlation between bacterial community and soil metabolism (Cheng et al., 2022). In the present study, the 15 metabolites related to disease resistance pathway that were significantly upregulated in disease resistance inbred lines were more significantly correlated with fungi. Where 13(S)-HOTrE, 16-Hydroxy hexadecenoic acid, LTB4, Dodecanedioic acid, 3-(3,4-dihydroxy-5-methoxy)-2-propenoic acid, and Narigenin had significant positive correlation with *Fusarium*. In addition, *Sphingomonas* was enriched in the resistant inbred line. This is consistent with the findings in rice (Matsumoto et al., 2021). Moreover, *Sphingomonas* showed significantly positive correlated with seven keystone metabolites (Figure 8A). These results indicated that *Sphingomonas* may have potential as a biocontrol agent against stalk rot.

## 5 Conclusion

To explore the relationship between plant and its microecology, root endophyte and rhizosphere soil microbiome were evaluated by high throughput sequencing, and UHPLC–MS was used to study changes in metabolisms. The results showed that there was different microbial community composition from different inbred lines in different growth stages. Co-occurrence network analysis showed that the pathogen *Fusarium* had the highest degree centrality and closeness centrality in the DR478. Moreover, metabolomics analysis showed that 4 main metabolic pathways were significantly enriched, and 15 metabolites were upgrade in resistant inbred line. Furthermore, microbes, especially fungi, also were related to these 15 metabolites. *Sphingomonas* may have potential as a biocontrol agent against stalk rot. Overall, these findings provide a theoretical basis for biocontrol of maize stalk rot.

## Data Availability

The datasets presented in this study can be found in online repositories. The names of the repository/repositories and accession number(s) can be found in the article/Supplementary material.

## Footnotes

^1^ www.majorbio.com

^2^ https://gephi.org/

## Supplementary material

SUPPLEMENTARY FIGURE S1 Bacterial **(A,C)** and fungi **(B,D)** rarefaction curves for all samples at a 97% OTU sequence similarity threshold.

SUPPLEMENTARY FIGURE S2 Venn diagram of unique and shared operational taxonomic units (OTUs) among samples. Numbers indicate the number of unique and shared bacterial **(A,E)** and fungal **(C,G)** OTUs. Microbial community pieplot of shared OTU among samples. Percentages indicate the proportion of specific OTU to shared bacterial **(B,F)** and fungal **(D,H)** OTUs.

SUPPLEMENTARY FIGURE S3 Relative abundances of dominant microbial taxonomic groups in all samples. **(A)** Dominant bacterial phyla in rhizosphere soil; **(B)** Dominant fungal phyla in rhizosphere soil; **(C)** Dominant bacterial phyla in root endophyte; **(D)** Dominant fungal phyla in root endophyte.

SUPPLEMENTARY FIGURE S4 Principal component analyses of microbial communities in different samples. **(A)** Rhizosphere soil bacterial community; **(B)** rhizosphere soil fungal community; **(C)** root endophyte bacterial community; **(D)** root endophyte fungal community.

SUPPLEMENTARY Table S1 Shared OTU table in all samples.

SUPPLEMENTARY Table S2 Differences of top 5 bacterial phyla among different compartments.
