# Supplementary figures and images for: Root-associated microbial diversity and metabolomics in maize resistance to stalk rot
Source: Front Microbiol. 2024 Dec 12;15:1468627. doi: 10.3389/fmicb.2024.1468627 (PMC11669678; doi:10.3389/fmicb.2024.1468627)

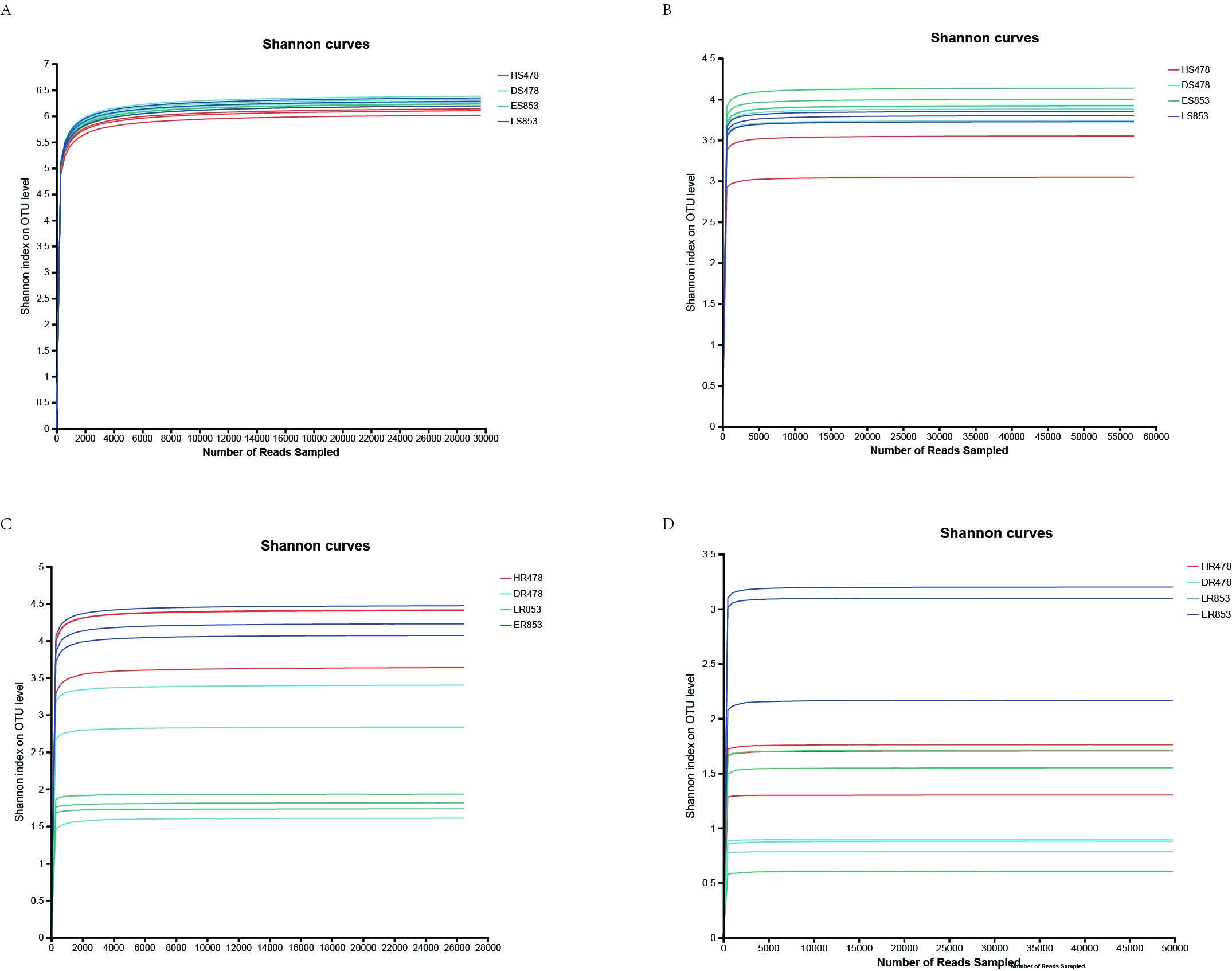

Supplement: SUPPLEMENTARY FIGURE S1 — Bacterial (A,C) and fungi (B,D) rarefaction curves for all samples at a 97% OTU sequence similarity threshold. [file Image_1.JPEG]

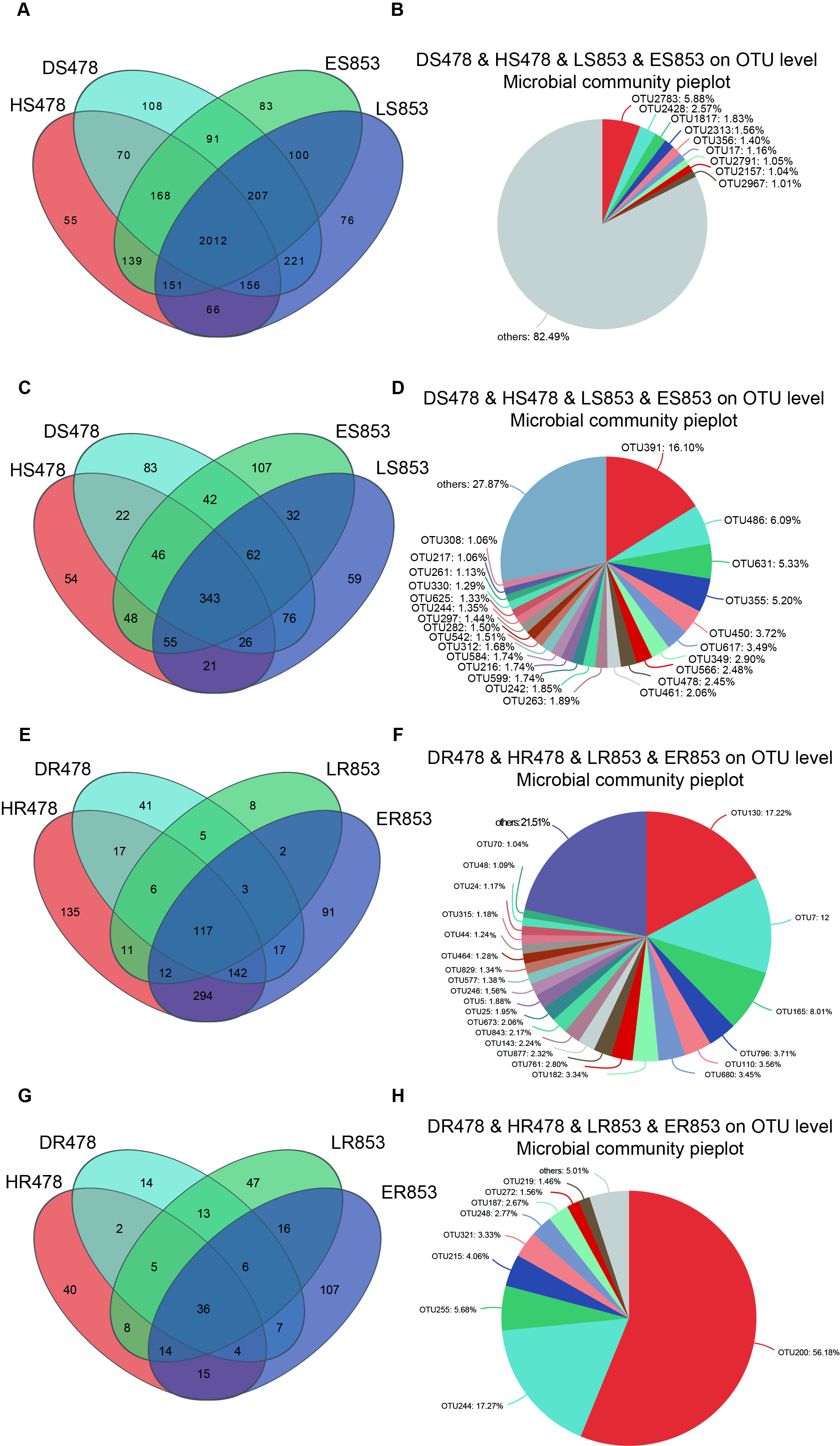

Supplement: SUPPLEMENTARY FIGURE S2 — Venn diagram of unique and shared operational taxonomic units (OTUs) among samples. Numbers indicate the number of unique and shared bacterial (A,E) and fungal (C,G) OTUs. Microbial community pieplot of shared OTU among samples. Percentages indicate the proportion of specific OTU to shared bacterial (B,F) and fungal (D,H) OTUs. [file Image_2.JPEG]

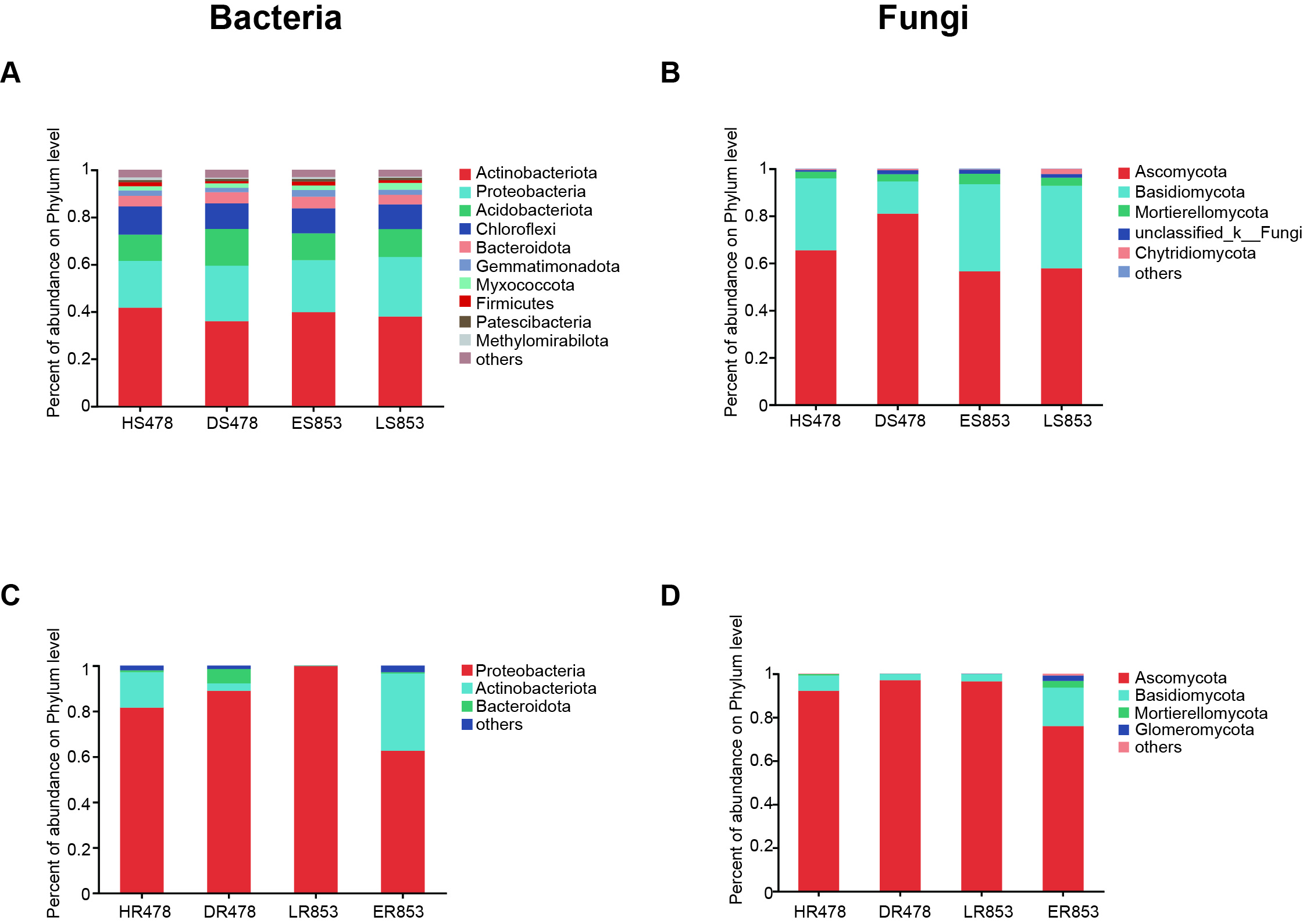

Supplement: SUPPLEMENTARY FIGURE S3 — Relative abundances of dominant microbial taxonomic groups in all samples. (A) Dominant bacterial phyla in rhizosphere soil; (B) Dominant fungal phyla in rhizosphere soil; (C) Dominant bacterial phyla in root endophyte; (D) Dominant fungal phyla in root endophyte. [file Image_3.JPEG]

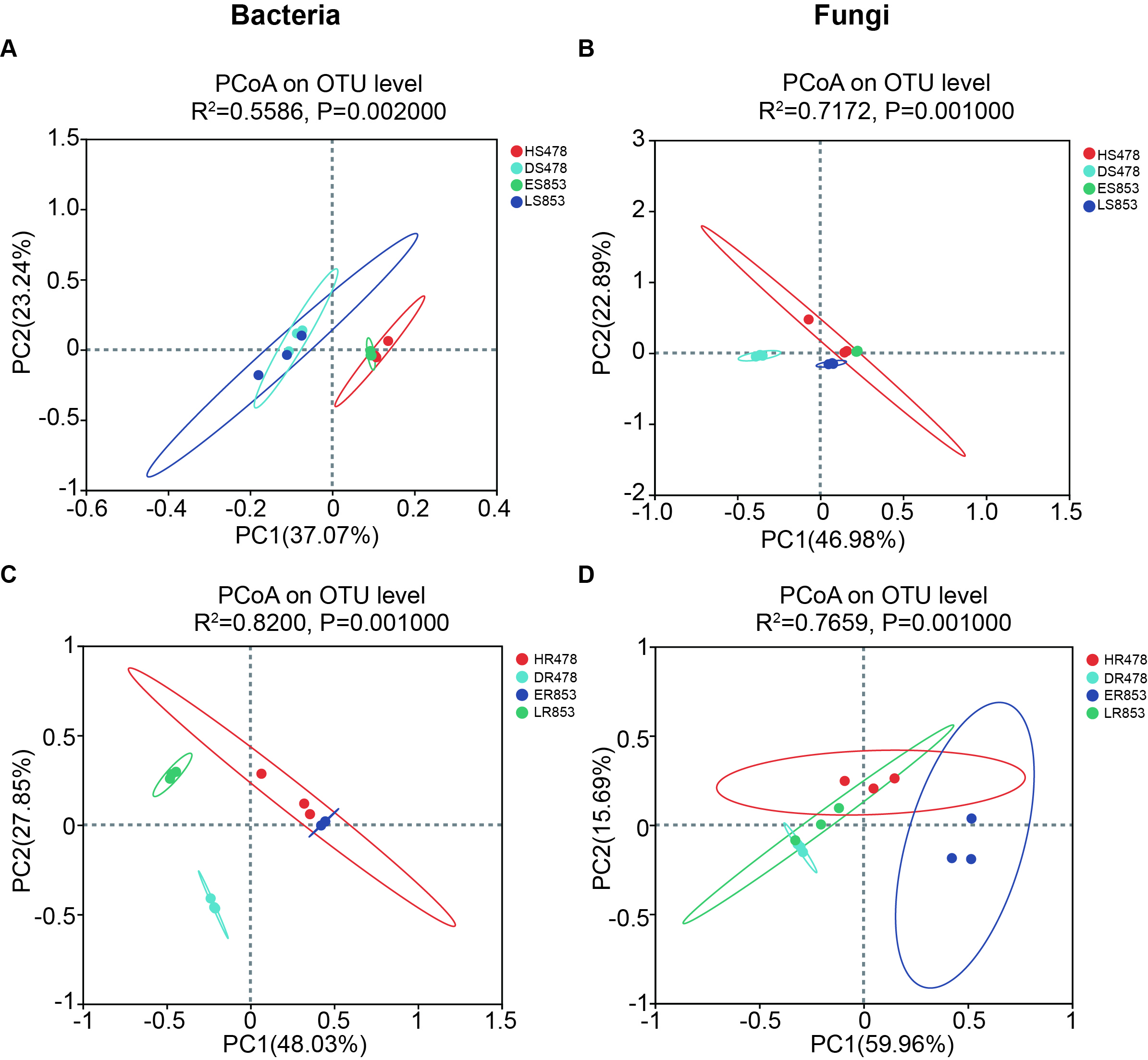

Supplement: SUPPLEMENTARY FIGURE S4 — Principal component analyses of microbial communities in different samples. (A) Rhizosphere soil bacterial community; (B) rhizosphere soil fungal community; (C) root endophyte bacterial community; (D) root endophyte fungal community. [file Image_4.JPEG]
